# Reassessing human MHC-I genetic diversity in T cell studies

**DOI:** 10.1038/s41598-024-58777-2

**Published:** 2024-04-04

**Authors:** Roderick C. Slieker, Daniël O. Warmerdam, Maarten H. Vermeer, Remco van Doorn, Mirjam H. M. Heemskerk, Ferenc A. Scheeren

**Affiliations:** 1 Department of Cell and Chemical Biology, Leiden University Medical Center, Leiden, The Netherlands; 2 Leiden Center for Computational Oncology, Leiden University Medical Center, Leiden, The Netherlands; 3 Centre for Future Affordable & Sustainable Therapy Development (FAST), The Hague, The Netherlands; 4 Department of Dermatology, Leiden University Medical Center, Leiden, The Netherlands; 5 Department of Dermatology, Netherlands Cancer Institute, Amsterdam, the Netherlands; 6 Department of Hematology, Leiden University Medical Center, Leiden, The Netherlands

**Keywords:** Cancer, Immunology, Medical research

## Abstract

The Major Histocompatibility Complex class I (MHC-I) system plays a vital role in immune responses by presenting antigens to T cells. Allele specific technologies, including recombinant MHC-I technologies, have been extensively used in T cell analyses for COVID-19 patients and are currently used in the development of immunotherapies for cancer. However, the immense diversity of MHC-I alleles presents challenges. The genetic diversity serves as the foundation of personalized medicine, yet it also poses a potential risk of exacerbating healthcare disparities based on MHC-I alleles. To assess potential biases, we analysed (pre)clinical publications focusing on COVID-19 studies and T cell receptor (TCR)-based clinical trials. Our findings reveal an underrepresentation of MHC-I alleles associated with Asian, Australian, and African descent. Ensuring diverse representation is vital for advancing personalized medicine and global healthcare equity, transcending genetic diversity. Addressing this disparity is essential to unlock the full potential of T cells for enhancing diagnosis and treatment across all individuals.

## Introduction

The MHC-I system is a family of proteins expressed on the surface of cells and is involved in the recognition and presentation of peptides to the immune system. It consists of a polymorphic heavy chain, a constant light chain called Beta-2 Microglobulin (β2M) and an 8–13 amino-acid peptide ligand^1,2^. The peptide binding groove of MHC-I heavy chain accommodates these peptides, and the properties of the pockets within the groove are important for peptide presentation. MHC-I comprises three major Human Leukocyte Antigen (HLA) families: HLA-A, HLA-B, and HLA-C, each consisting of numerous alleles. Among these, HLA-A and HLA-B exhibit the greatest diversity, while HLA-C shows less variation^3^. HLA-C is associated with multiple additional receptors, such as Killer Immunoglobulin-like Receptors (KIRs) that can be expressed on T cells, adding complexity to the system and complicating diagnostics of HLA-C restricted T cells^4^. Consequently, these complexities contribute to the tendency to overlook HLA-C in research studies. The diversity in the MHC-I heavy chain directly influences peptide presentation by altering the properties of the pockets in the peptide binding groove. This genetic polymorphism results in changes in the size, shape, and electrostatic properties of the pockets, which in turn affect the binding affinity and specificity of the MHC-I molecule for different peptides, thereby directly influencing the peptide repertoire. Although peptides may have overlap between similar MHC-I heavy chains, each allele has an unique repertoire of peptides^5^. Hence, understanding that while the overall structure of MHC-I remains largely consistent, the specific composition of alleles and variations in anchor residues give rise to unique peptide-binding specificities within each population, resulting in exceptionally high sequence diversity. This leads to an extensive array of alleles, totalling over 37,000 variants, some of which are exclusive to particular ancestral populations^6–9^. This diversity in MHC-I alleles enhances the population's ability to mount effective immune responses against a given pathogen by increasing an individual’s chance of eliciting a suitable immune defence. Thus, MHC-I diversity helps to protect against pandemics. For example, the HLA-B*15:01 allele is more prevalent in Southeast Asian populations compared to European populations, highlighting the geographic variability in HLA alleles^8^. However, this diversity also represents a challenge in the biomedical domain due to its potential to reinforce existing disparities, potentially leading to unequal healthcare based on an individual’s MHC-I alleles.

The relevance of MHC-I alleles in diagnostics and immunotherapy has surged in recent years, offering potential applications in disease diagnosis and treatment^10–16^. The groundwork for this technology was laid in 1996 with the introduction of recombinant MHC-I technology, enabling the visualization of antigen-specific cells^17^. This methodology involves the synthesis of MHC-I molecules via synthetic DNA sequences in a laboratory setting. These recombinant soluble MHC-I monomers, complexed with specific peptides, are then multimerized and fluorescently labelled, commonly referred to as tetramers or multimers. These multimerized peptide MHC-I complexes play a crucial role in immune response monitoring by facilitating the specific binding of antigen-specific T cells, allowing the visualization, and tracking of T cell responses over time. They have applications in diagnostics, aiding in the identification of antigen-specific T cells and differentiating between vaccination and natural infection based on pathogen protein coverage. Recent studies have extensively investigated T cells in COVID-19 patients^18–20^. Furthermore, recombinant MHC-I technology has significantly impacted cancer immunotherapy. This technology leverages the fundamental link between TCR and peptide-HLA complexes, enabling the precise targeting of cancer cells. Customized recombinant peptide MHC-I complexes help identify specific cancer antigens, paving the way for personalized treatment strategies. Additionally, they play a pivotal role in TCR-based therapies by facilitating the precise targeting of cancer cells by engineered T cells. Clinical trials utilizing HLA-restricted TCRs are currently underway, either by introducing TCRs into patient T cells or employing recombinant TCR fusion proteins fused to anti-CD3, resulting in bispecific T cell engagers^10–14^. These therapies are designed to target specific immune responses mediated by predetermined HLA alleles. Moreover, recombinant MHC-I technology serves as a peptide-specific platform for inducing T cell proliferation in an antigen-specific manner^21,22^. In summary, the versatility and effectiveness of recombinant MHC-I technology position it as a cornerstone in the ongoing battle against cancer through immunotherapy.

Given the extensive polymorphism among HLA genes and their connections to population genetics, we set out to investigate whether there are biases in the HLA alleles studied in medical research. Our analysis focused on the utilization of MHC-I alleles in both clinical and preclinical publications, particularly those related to COVID-19 and clinical trials focused on TCR-based immunotherapies. Our findings reveal a notable underrepresentation of alleles found in people from Asian, Australian, and African descent, suggesting a widespread allele bias in medical research and clinical therapeutic development.

## Results

This study conducted a comprehensive search for articles published between August 2020 and April 2023, focusing on T-cell research related to Severe Acute Respiratory Syndrome Coronavirus 2 (SARS-CoV-2) and Human Leukocyte Antigen Class-I (HLA-I), representing the human MHC-I, aiming to assess the breadth of HLA alleles studied in medical research. SARS-CoV-2 was chosen as a model infection due to the extensive utilization of MHC-I technology in research on the immune response to the virus and its global impact, affecting all continents and countries. Out of 615 articles identified, 74 were included, focused on allele-specific analyses, in specific MHC-I technology. In the 74 studies we considered, individual epitopes, the specific portion of the peptide sequence that is recognized and bound by the MHC-I molecule, against COVID-19 were determined by using mono-allelic MHC-I multimer qualitative binding (Table S1, Fig. S1). A total of 22 unique MHC-I alleles were used with epitopes of SARS-CoV-2. MHC-I allele frequencies have a widespread variation across human populations, however the geography on the distribution of MHC-I alleles resembles geographical location of populations^23^. For this reason, we analysed the frequencies of the MHC-I allele usage in literature in relation to the frequency of occurrence in a specific continental or intra-continental group. The following continental and intra-continental groups were included in our analysis: South Asia, North-East Asia, South-East Asia, Sub-Saharan Africa, Oceania, North Africa, Western Asia, South and Central America, North America, and Europe.

**Figure 1:**
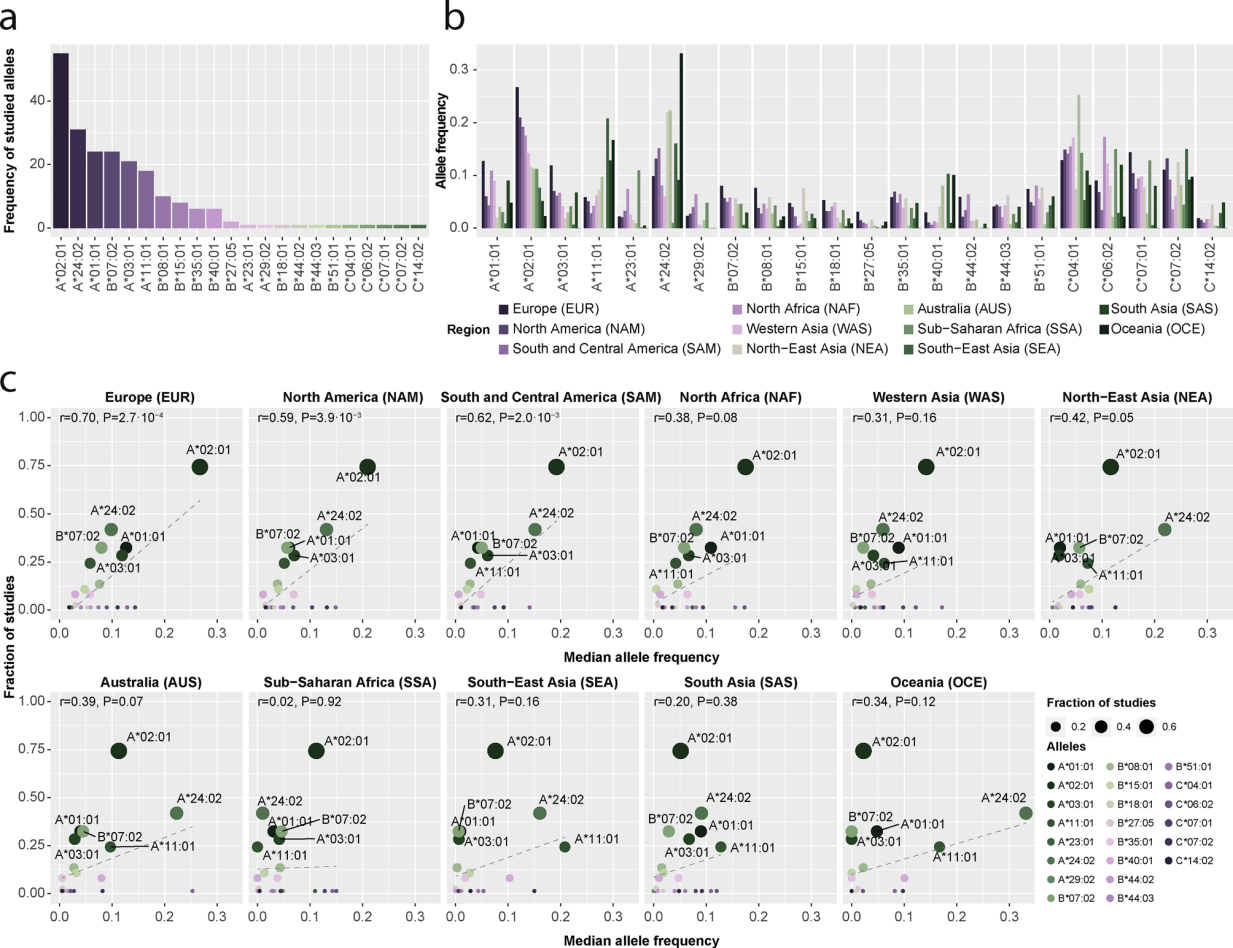
Comparison of studied allele frequencies across geographical locations. (**a**) Frequency of the alleles across included COVID-19 studies. X-axis studied alleles, Y-axis, number of articles. (**b**) Frequency of studied alleles across geographical regions. X-axis, allele; y-axis, allele frequency as reported on Allele Frequency Net Database (https://www.allelefrequencies.net/pops.asp) Colours indicate the geographical region. All alleles showed a significant difference in allele frequency across geographical regions (P < 3.9 10^−5^, ANOVA). (**c**) Correlation between fraction of studies and median allele frequency for each allele in a specific geographical location. X-axis, median allele frequency; y-axis and dot size, fraction of studies. Numbers indicate the Pearson correlation between the median allele frequency and the fraction of studies with the corresponding P-value.

**Figure 2:**
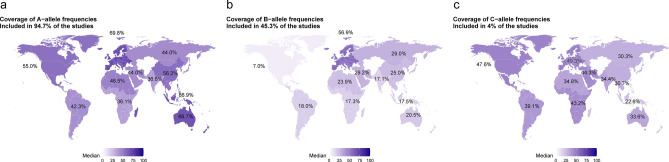
Coverage of alleles in studies investigated. Percentage in each continent represents the coverage of alleles. Different panels represent the different HLA alleles, which is HLA-A (**a**), HLA-B (**b**) and HLA-C (**c**). The colour indicates the percentage coverage of the alleles.

The HLA-A*02:01 allele was included in the majority of the studies, i.e. 55 of the 74 studies included for analysis (74.3%), followed by HLA-A*24:02 (N = 31, 41.9%), HLA-A*01:01 (N = 24, 32.4%), HLA-B*07:02 (N = 24, 32.4%), HLA-A*03:01 (N = 21, 28.4%, Fig. 1a). For studied alleles, there was a difference in frequency across geographical populations (Fig. 1b). Indeed, there was a strong positive correlation between the frequency of alleles used across studies and the allele frequency in Europe (r = 0.70, P = 2.7·10^−4^), North America (r = 0.59, P = 3.9·10^–3^) and South and Central America (r = 0.62, P = 2.0·10^−3^) (Fig. 1c**)**. We observed a weak correlation or absent correlation for North Africa (r = 0.38, P = 0.08), Western Asia (r = 0.31, P = 0.16), North-East Asia (r = 0.42, P = 0.05), Australia (r = 0.39, P = 0.07), Sub-Saharan Africa (r = 0.02, P = 0.92), South-East Asia (r = 0.31, P = 0.16), South Asia (r = 0.20, P = 0.38) and Oceania (r = 0.34, P = 0.12). Overall, the HLA-A alleles were the most studied alleles (Fig. 2a) and showed the best coverage for Europe (69.8%), while the lowest coverage for Sub-Sharan Africa (36.1%). For the HLA-B alleles, a similar pattern was observed with a high frequency in Europe but low in Sub-Sharan Africa. The B-alleles particularly showed a low coverage in North America (Fig. 2b), but this was mainly driven by the low frequency in Mexico (5.1%) versus USA-based studies (41.2%, Fig. 2b). HLA-C alleles were not extensively researched, accounting for only 4% of the studies, despite their relative high population coverage across continents (Fig. 2c).

Our analysis was repeated based on an independent dataset obtained from a systematic review of T-cell epitopes defined from the proteome of SARS-CoV-2, describing 1349 MHC-I epitopes^20^. This validation in a second COVID-19 dataset obtained from this systematic review of T-cell epitopes is essential to ensure the robustness and generalizability of the initial analysis and to minimize the risk of bias. The acquired alleles were not restricted to MHC-I technology, and this systematic review provides a description and explanation of the diverse range of technologies utilized. This analysis gave a similar distribution of investigated alleles was observed, with most studies including HLA-A*02:01 (N = 34, 79.1%), followed by HLA-A*24:02 (N = 16, 37.2%) and HLA-A*01:01 (N = 14, 32.6%). Again, the highest correlations were observed for Europe followed by the Americas (Fig. S3).

**Figure 3:**
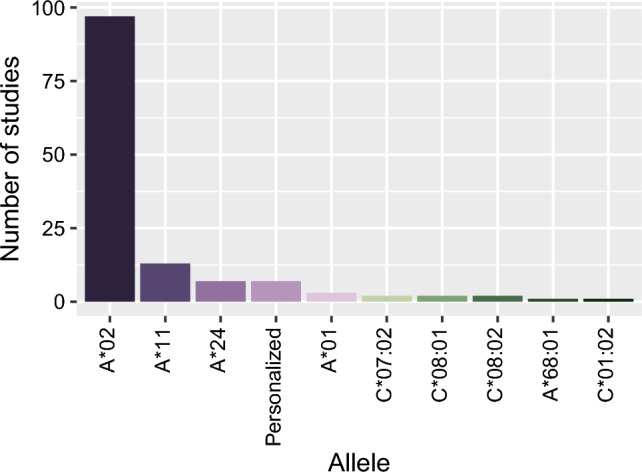
Coverage of alleles in TCR-based immunotherapies. Number of studies and the HLA-I alleles used in TCR-based clinical trials is shown. Data consists of 118 TCR-transfer studies and eight recombinant TCR studies. The personalized TCR-transfer studies (N = 7) were all TCR-transfer clinical trials. Out of the 97 studies that reported HLA-A*02, 58 reported A*02:01 specifically while the others A*02 only. X-axis, allele; y-axis, number of studies.

Next, we analysed the clinical trials that make use of TCR-based immunotherapy. TCRs can only recognize and bind to a specific peptide presented by a particular MHC-I allele. Therefore, TCR-based immunotherapy treatments are designed to target specific peptides presented by one predetermined MHC allele^24,25^. For this reason, TCR-based immunotherapies are HLA-restricted therapies. By examining these clinical trials, we can assess for which specific HLA alleles therapies are designed. This analysis can help to guide future research and clinical development efforts towards more personalized and effective treatments for patients with specific genetic backgrounds. Using the clinicaltrials.gov website we found 126 studies in which TCR transfer was clinically used (N = 118) or recombinant TCR fusion proteins were used (N = 8). The latter are all HLA-A2 restricted. The allele coverage of these TCRs showed clear over-representation of HLA-A2 in these clinical trials (Fig. 3). Seven studies mention that personalized TCRs will be developed, however the coverage of alleles was not mentioned and thus it was difficult to determine how large the allele diversity will be in these clinical studies. The focus on HLA-A*2, and in specific A*02:01, means that a large population is excluded in these clinical trials. For example, within the American population, people with an African American or Asian genetic descent will have an almost 50% lower chance to enrol in these TCR-based immunotherapy trials^26,27^.

## Discussion

These results demonstrate that the preclinical and clinical analyses of antigen-specific T cell diagnostics and the clinical development of HLA-I restricted therapies, such as TCR-based immunotherapies, show an underrepresentation of people with an Asian, African, Australian, and Oceanian descent. We provide data for both the COVID-19 outbreak and TCR-based therapies. However, we believe that the underrepresentation of specific HLA alleles is not confined to only these two fields; rather, they serve as examples representing the broader scope of the field. Within the clinical setting there is a strong bias towards the use of HLA-A*2. This raises concerns about the inclusivity and generalizability of findings within the preclinical and clinical analyses of antigen-specific T cell research and diagnostics that rely on MHC-I technologies. Conversely, populations with European and American (North, South, and Central) descent exhibit robust representation in these T cell-focused investigations and clinical trials.

This lack of MHC-I allele diversity within T cell research and clinical setting is multifaceted, rooted in historical, methodological, and systematic factors. Historically, since the HLA-A02:01 allele is present in 50% of the Caucasian population, the initial analyses of T cell responses have been focused on the HLA-A02:01 allele^17,28^. Early reagents, such as HLA-I restricted T cell clones and later recombinant HLA-A*02:01 were focused on the European population resulting in a skewed research field. Additionally, peptide affinity predictions for specific alleles rely on data obtained through wet lab experiments, such as mass spectrometry based ligandome^29^. The accuracy of these predictions improves with the availability of more data. Less characterized alleles have a poorer performance for the in-silico peptide predictions^30^. As a result, researchers gravitate toward well-characterized alleles, reinforcing a feedback loop that perpetuates the imbalance. Increased availability of diverse recombinant MHC-I alleles in combination with high-quality peptide databases needed for high-accuracy in silico predictions would enhance the diversity in biomedical research needed for T cell analyses in a diverse population. Additionally novel technologies allowing HLA-unbiased TCR are also developed and very important in ensuring diverse HLA representation^31,32^.

The ramification of this underrepresentation involves critical aspects. Firstly, the COVID-19 vaccine and clinical trial landscapes exhibited overrepresentation of white non-Hispanic participants, mirroring trends in cancer immunotherapy research^33,34^. Given the influence of MHC-I allele diversity on disease outcome of pathogens such as SARS-CoV-2, vaccine responses, and effectiveness, the bias hampers generalizability and obstructs insights into diverse population responses to interventions^35–38^. In the context of COVID-19, variations in HLA genes influence an individual's susceptibility to the virus, severity of the disease, and response to treatments or vaccines^35,39–41^. Therefore, investigating the impact of HLA on patients with COVID-19 is essential for both clinical management and public health strategies.

Secondly, while pivotal scientific breakthroughs hold importance, the next step entails integrating human genetic diversity into research paradigms. Within the field of genome editing, genetic data from people with a diverse ancestry is essential to determine the CRISPR off-targets, and thereby assess safety and efficacy^42^. Originally the human genome project consisted of 70% of only one person with a blended ancestry. The remaining 30% came from 19 individuals of European ancestry^43^, resulting in a very limited genomic diversity. Although currently the vast majority of genomics studies have been conducted in individuals of European descent, the human genome field is now taking the lead by rapidly increasing the number of reference genomes from individuals with diverse ancestry^44,45^. This inclusion of genomic diversity is not only essential for genome editing but ensures that the benefits of genomic medicine are accessible to all. Just as the inclusion of diverse genetic ancestry is pivotal in genome editing, genomic medicine's broad applicability hinges on embracing genetic diversity. Incorporating genetic variability needs to become a cornerstone in the progress of immune diagnostics and immunotherapy, mirroring the trajectory of genomic medicine.

The significance of MHC-I alleles in COVID-19 outcomes underscores the necessity of comprehensive representation in COVID-19 T-cell studies, compelling the inclusion of individuals with underrepresented genetic makeup. Similarly, the overrepresentation of HLA-A*2 in TCR-based immunotherapies should intensify the urgency for equitable representation. The over-representation of the HLA-A*2 allele impedes the versatility of TCR application and reveals a skewed MHC-I allele representation in therapeutic contexts. Bridging this gap requires increased awareness and strategic funding. An example of such endeavour is the Cancer Grand Challenge of 2023 (https://cancergrandchallenges.org/challenges), which addresses disparities in cancer research across diverse populations.

Beyond the scientific field, the implications of this underrepresentation extend to inequities in therapy development and healthcare availability, demonstrating the necessity to engage diverse communities in biomedical science. The ethical goal to include MHC-I genetic diversity aligns with the scientific goal, as the biological associations of specific MHC-I alleles underscore the complexity that demands comprehensive understanding. The absence of equal representation poses formidable barriers to advancing T cell-based therapies in the era of personalized medicine.

## Material and methods

### Article inclusion

Articles published in peer-reviewed journals before April 2023 were included. Articles were identified using the following search term:

(“SARS-CoV-2” OR “COVID-19”) AND “T-cell” AND (“tetramer” OR “multimer”) AND “HLA-A”.

We used specifically Google Scholar given that the use of tetramers is often not described in the abstract. Indeed, the search term above yielded only 6 results in PubMed versus 615 articles on Scholar. Out of the 615 articles, 74 were suitable for inclusion, given that 7 were non-English, 323 did not report on COVID-19 but only mentioned it in the text, 177 articles were different types of articles including reviews and opinions and 34 were on a COVID-19-related topic (Fig. S1, Table S1). Regarding the latter, we focused on allele-specific analyses, and we did not include experiments, in which there was no active discrimination between the 6 different MHC-I alleles expressed in one person. Clinical trials were obtained from the website https://clinicaltrials.gov/. Trials were identified using the following search term: “TCR-T cell” AND “TCR therapy” AND “TCR-CD3 therapy” AND “TCR”. We selected only TCR transfer trials and trials that used a recombinant TCR fusion protein, such as Tebentafusp. Studies were included also when they only reported the antigen, for example A*02.

### Allele frequencies in different populations

Allele frequencies in different geographic locations were obtained from the Allele Frequency Net Database (http://www.allelefrequencies.net/, access date July 2023^8^). Country of each study was assigned to regions as defined by the Allele Frequency Net Database (https://www.allelefrequencies.net/datasets.asp).

### Replication set

The analysis was repeated in an independent dataset obtained from a systemic review of T-cell epitopes defined from the proteome of SARS-CoV-2, describing 1349 MHC-I epitopes^20^. We only included epitopes that were predicted for one specific allele and excluded epitopes for potentially two or more different MHC-I alleles. These epitopes were functionality tested using the following assays: ELISA, HTMA, multimer staining, cytotoxicity, AIM, ICS, ELISPOT and, or proliferation. Alleles investigated in each study included in the systematic review were extracted and frequencies of alleles across studies determined.

### Statistical analysis

Total coverage of a geographical region was calculated as described previously^46^. For each study on The Allele Frequency Net Database, we calculated the sum of all identified MHC-I class allele frequencies. When the sum of the allele frequency exceeded one, observed allele frequencies were scaled based on the sum value. When below one, it was assumed that there was an additional unmeasured allele. Next, alleles that were included in articles were summed to get a measure of the coverage of a population by the current studies on COVID-19. Median allele frequencies were calculated for each region, by taking the median allele frequency of an allele across studies. Median allele frequencies for each country are given in Table S2. The median allele frequency per region was plotted against the fraction of studies that studied a specific allele. Correlations between study frequency and allele frequency were determined based on Pearson correlation and a P-value below 0.05 was considered significant. Figures were produced using R4.3.0 in combination with *ggplot2* (v3.4.3) and *patchwork* (v1.1.3). Geographical maps were produced with ggplot2 using the map data function; https://ggplot2.tidyverse.org/reference/map_data.html

### Supplementary Information

Supplementary Figures.

Supplementary Table S1.

Supplementary Table S2.

## Data Availability

All data generated or analysed during this study are included in this published article and its supplementary information files.

## Code availability

All R code used in the current study is available from GitHub: https://github.com/roderickslieker/HLA_Disparity
