# Supplementary material for: Reassessing human MHC-I genetic diversity in T cell studies
Source: Sci Rep. 2024 Apr 4;14:7966. doi: 10.1038/s41598-024-58777-2 (PMC10995142; doi:10.1038/s41598-024-58777-2)
Supplement: Supplementary file 1 — Supplementary Figures. [file 41598_2024_58777_MOESM1_ESM.docx]

**Supplemental Figures**

**Figure S1. Selection of articles in the current study.**


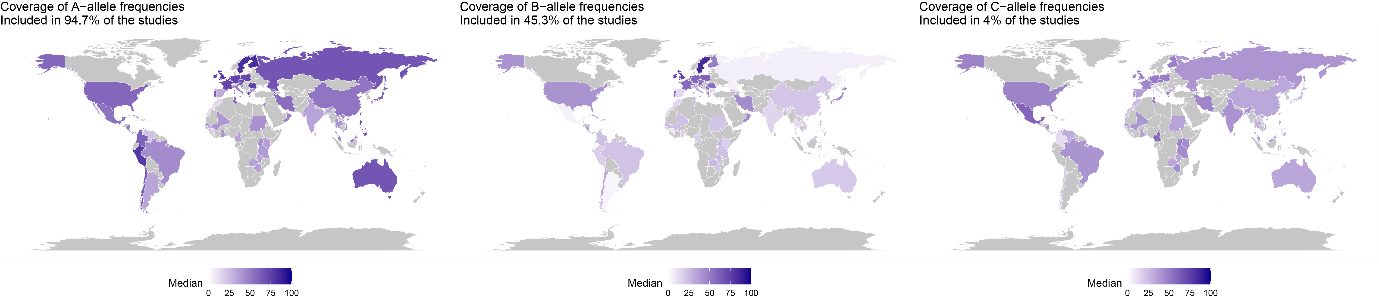


**Figure S2 Coverage of alleles in studies investigated.** Blue color indicates the coverage of alleles in each country. White, low coverage, dark blue high coverage, gray no data.

**
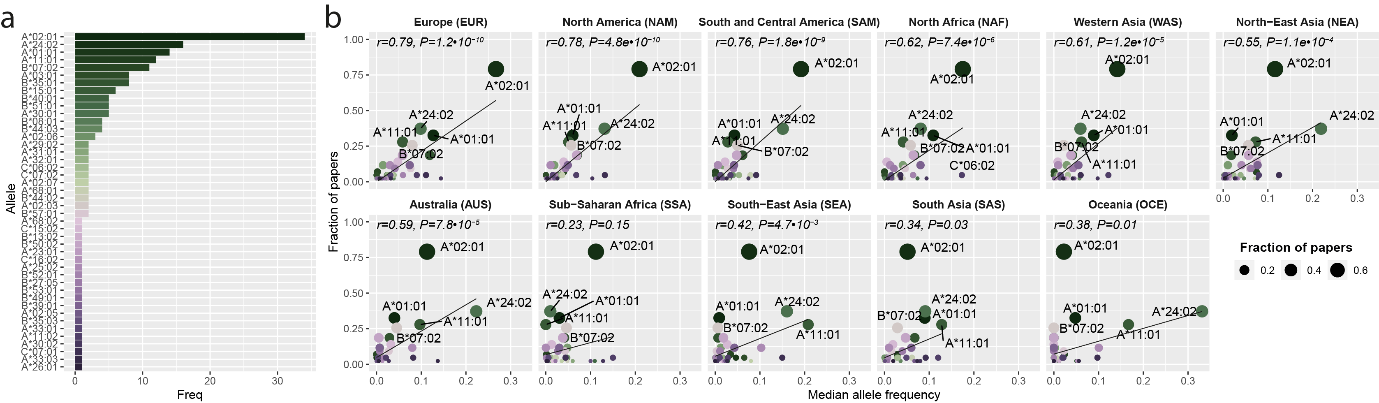
**

**Figure S3 Comparison of studied allele frequencies across geographical locations based on the systematic review of Jin *et al*. a** Frequency of the alleles across included studies. X-axis number of papers; y-axis, studied alleles. **b** Correlation between fraction of papers and median allele frequency for each allele in a specific geographical location.
